# Pazopanib is an active treatment in desmoid tumour/aggressive fibromatosis

**DOI:** 10.1186/2045-3329-3-13

**Published:** 2013-11-26

**Authors:** Juan Martin-Liberal, Charlotte Benson, Heather McCarty, Khin Thway, Christina Messiou, Ian Judson

**Affiliations:** 1 The Royal Marsden Hospital, Sarcoma Unit, Fulham Road, SW3 6JJ London, UK; 2 Belfast City Hospital, Lisburn Road, BT9 7AB Belfast, UK

**Keywords:** Aggressive fibromatosis, Desmoid tumour, Imatinib, Pazopanib, PDGFR, Pegylated doxorubicin, Sorafenib, VEGFR

## Abstract

**Background:**

Desmoid tumours/aggressive fibromatosis (DT/AF) are infrequent soft-tissue neoplasms. They usually behave as indolent diseases. However, they may grow locally infiltrating or compressing adjacent structures. The role of local treatment is limited and only a few drugs have shown activity.

**Cases presentation:**

We report the outcome of two patients affected by progressive DT/AF treated with the angiogenesis inhibitor pazopanib in two different institutions. Both patients achieved dramatic improvement in their symptoms and radiological signs of response. The clinical benefit lasted for more than 1 year and it is still ongoing.

**Conclusions:**

Pazopanib is an active treatment in DT/AF. It is the first time this has been reported.

## Background

Desmoid tumours/aggressive fibromatosis (DT/AF) are rare soft-tissue neoplasms that usually arise from the abdomen although extremities are also a common site of presentation [1,2]. They may be associated with genetic conditions such as Gardner’s syndrome [3]. Patients’ survival is usually good as DT/AF lack the capacity to metastasize although anatomical disease site is important. Interestingly, the treatment paradigm has changed in the last decade. Aggressive upfront approaches are now under debate since nearly 50% of patients have relatively indolent disease [4]. Thus, surveillance at initial presentation is the current standard of care in most centres. However, it may become a symptomatic disease as it can grow locally infiltrating or compressing adjacent structures [5].

Surgery is one treatment choice when technically feasible however rates of recurrence and post-treatment morbidity are high [6]. Radiotherapy is also an option but there are real concerns surrounding late effects including development of second malignancies which is important to consider given the young age of most patients. When local treatment with curative intent is not achievable, systemic treatment should be considered. A widely accepted strategy in first-line setting is hormone treatment, based on the relatively frequent overexpression of oestrogen receptors, used in combination with nonsteroidal anti-inflammatory drugs (NSAIDs) [7,8]. If this treatment proves ineffective, cytotoxic chemotherapy is a valid alternative. Pegylated liposomal doxorubicin has shown signs of activity although it is not without toxicity [9]. Several targeted agents have been recently assessed with promising results. The, tyrosine-kinase inhibitor imatinib has been proven to have activity in phase II studies [10,11]. Also, recent encouraging data with the antiangiogenic drug sorafenib have been published where the rate of clinical benefit was very high (70%). In addition, partial response (PR) and stable disease (SD) rates were 25% and 70% respectively. Furthermore, 92% of patients showed features of increased tumour fibrosis and loss of cellularity as demonstrated by an early change in MRI T2 signal [12]. These data support further investigation into the role of antiangiogenic agents in DT/AF.

Pazopanib is one of the latest antiangiogenic drugs developed. It has recently been approved by the U.S. Food and Drug Administration (FDA) and by the European Medicines Agency (EMA) for the treatment of advanced renal cancer and soft-tissue sarcomas (STS). We present here the first report of clinical activity of pazopanib in DT/AF. These encouraging results might be the initial step in the development of a new effective therapy this challenging disease.

## Cases presentation

This is the report of the clinical outcome of two patients affected by unresectable DT/AF treated with pazopanib in two different UK institutions: The Royal Marsden Hospital, London and Belfast City Hospital, Belfast. Data were retrospectively collected and imaging was reviewed by a Sarcoma Unit radiologist from The Royal Marsden Hospital.

### Patient 1

Patient 1 is a female who presented at the age of 42 years with a painful mass in the right axilla. A biopsy showed features consistent with DT/AF. The patient did not have personal or family history of Gardner’s syndrome or colonic polyps. Surgical resection was performed in two occasions but residual disease remained. As the pain in the axilla increased, treatment with tamoxifen 40 mg once daily (od) was initiated one year after the second surgery. However, there was an increase in the size of the mass and worsening of symptoms in the form of restricted movement and neuropathic pain, so pegylated liposomal doxorubicin was advised. Unfortunately, chemotherapy had to be stopped after one cycle due to an allergic reaction. Subsequent treatment with methotrexate and vinblastine was given. Symptoms were stabilized although the patient experienced disabling toxicities such as severe constipation and neuropathy. After five months, an MRI showed progressive disease (PD), the tumour measuring 15 × 5.9 cm.

**Figure 1:**
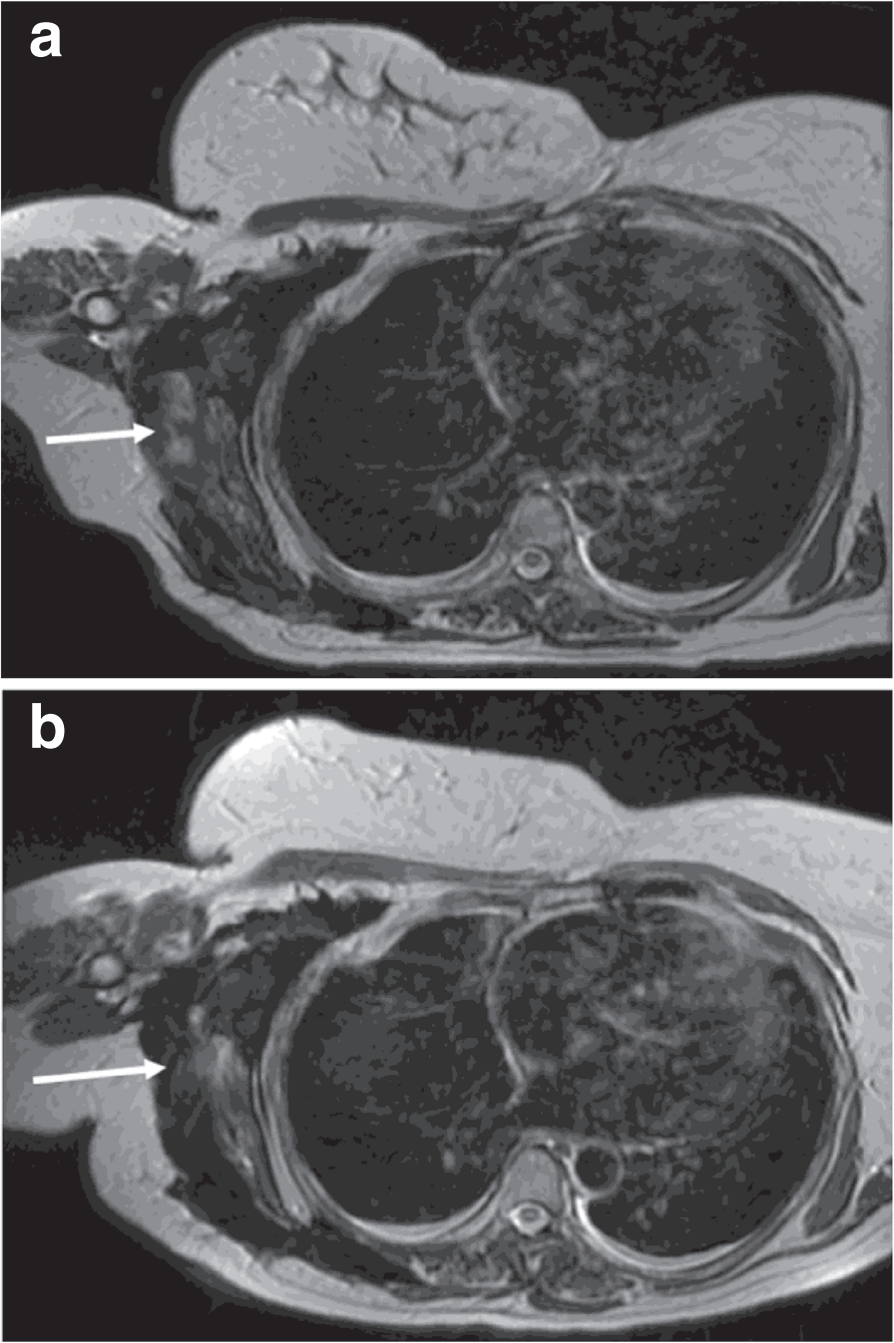
**Axial T2 weighted MRI images of the thorax at baseline (a) and following 10 months of pazopanib (b).** A large plaque of fibromatosis encases the lateral right hemithorax extending into the axilla. Although the disease remained dimensionally stable the lateral component (arrows) demonstrated T2 signal drop from intermediate to low indicating a reduction in cellularity.

At this point, treatment with pazopanib 800 mg od was started. Shortly afterwards, the patient developed hypertension, dysgeusia and mild palmar erythema so the dose was reduced to 600 mg od. After two months, the patient reported a significant improvement in her pain. Analgesic requirements were dramatically reduced and an MRI showed no changes in size but a significant reduction in tumour T2 signal intensity, suggesting reduction in cellularity. Six months after starting pazopanib, a further dose reduction due to diarrhoea was needed (400 mg od). At that time, a second assessment MRI showed maintained SD and the drop in T2 signal intensity compared to baseline remained suggesting continued response (Figure 1). In the following months, further toxicities appeared: skin rash, peripheral neuropathy and worsening hypertension, dysgeusia and diarrhoea. These side effects led to a further dose reduction to 200 mg od and optimization of supportive treatment.

The patient is currently tolerating pazopanib without significant side effects. Furthermore, her tumour has remained stable in size but with T2 changes in MRI suggestive of response for nearly eighteen months. The clinical benefit has been very clear and in particular pain is well controlled, which was the main factor impairing the patient’s quality of life.

### Patient 2

Patient 2 is a female who presented at the age of 16 years with a painful left axillary mass. The histological analysis was consistent with DT/AF. The patient did not have a history of Gardner´s syndrome or colonic polyps. Incomplete resection was performed and for the following ten years the patient remained asymptomatic. After that time, she developed pain and impairment in the abduction of her left arm. An MRI confirmed tumour recurrence, with involvement of the triceps and inferior deltoid muscles. Surgery was not advised and the patient was started on systemic treatment with high dose NSAIDs; three months on MRI scan showed no significant changes and limitation in the abduction of the arm persisted. After one year of treatment, the patient became pregnant and treatment was discontinued. Unfortunately, shortly after the symptoms dramatically worsened. Pegylated liposomal doxorubicin was initiated when the patient was in the third trimester of her pregnancy but with no radiological nor symptomatic benefit. As there were continued significant limitations in arm movement, the patient was very keen to pursue further treatment. Therefore, tumour resection followed by radiotherapy was performed. After these procedures, the patient regained an almost complete range of movement in her arm.

One year after her last operation, an MRI showed two new sites of disease. These findings were associated with worsening of the pain in spite of NSAIDs. Tamoxifen, together with continuation of high dose NSAIDs, was then prescribed. Six months on, further tumour growth and worsening symptoms suggested that the treatment was ineffective.

**Figure 2:**
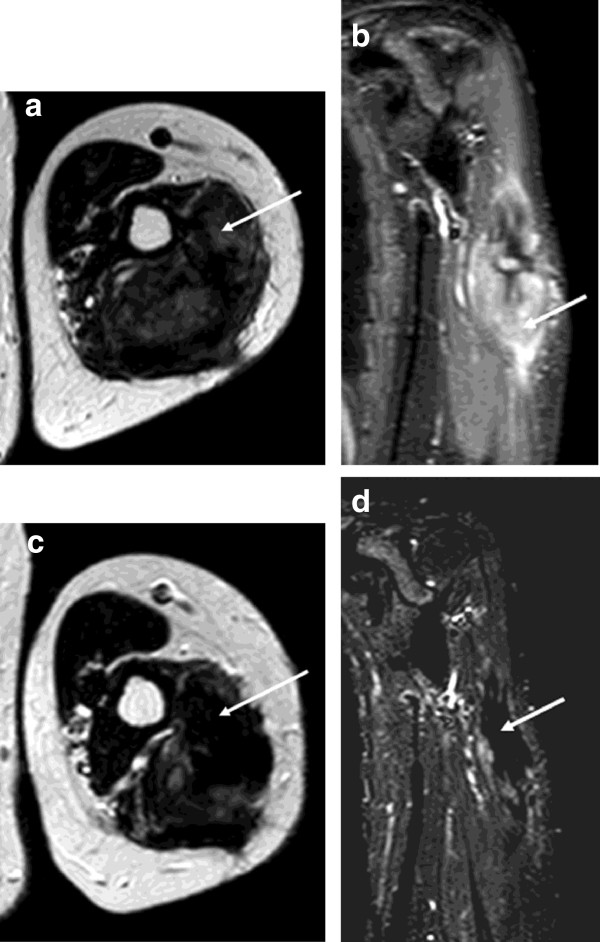
**Axial T2 weighted and coronal STIR MRI images of the proximal left arm at baseline (a and b) and following 11 months of pazopanib (c and d).** A large focus of fibromatosis expands the triceps muscle and following 11 months of treatment reduced in size from 10.2 cm in maximum craniocaudal dimension to 8.0 cm. Predominantly intermediate/high signal tissue (**a** and **b**, arrows) showed a marked reduction in signal (**c** and **d**, arrows) indicating diminished cellularity.

A new line of treatment with pazopanib 800 mg od was started. After two weeks of treatment, significant improvement of the pain was noticed. Moreover, the first assessment MRI after three months demonstrated reduction in tumour size (from 10.2 × 4.2 cm at baseline to 9 × 3.1 cm). Mild diarrhoea and fatigue were the only side effects. Subsequent MRIs showed further tumour shrinkage and reduction in T2 signal (Figure 2). In addition, mobility of patient’s left arm progressively improved. The patient remains on full dose of pazopanib without significant toxicities and with ongoing clinical and radiological benefit more than one year since starting pazopanib.

## Conclusions

This report demonstrates for the first time that pazopanib is an active treatment in DT/AF.

The lack of effective therapeutic options and its high morbidity make DT/AF a challenging disease. The promising results observed with sorafenib suggested that angiogenesis might play an important role in this condition [12]. Although DT/AF is not strictly considered a malignancy, the mechanisms that lead to uncontrolled monoclonal cellular proliferation and survival are similar to those in cancer [13]. Angiogenesis is one of the most important processes in carcinogenesis and its relevance in soft-tissue tumours has also been demonstrated [14]. Several studies confirm the key role of some of its effectors such as the vascular endothelial growth factor (VEGF) and the different isoforms of its receptor (VEGFR) [15-19]. Another crucial protein in the formation of new tumour vessels is the platelet-derived growth factor PDGF [20]. Interestingly, both sorafenib and pazopanib inhibit both VEGFR and the PDGF receptor (PDGFR) [21]. Conversely imatinib -a tyrosine-kinase inhibitor which also demonstrates promising activity in DT/AF [10,11]- has effects on PDGFR but not on VEGFR [22]. These data suggest that the efficacy of these drugs in DT/AF might be due to their antiangiogenic activity mediated by the inhibition of different key effectors.

Pazopanib is the first antiangiogenic drug that has shown successful results in a phase III trial in STS [23]. We demonstrate in this report is that it is also active in DT/AF. Furthermore although it has side effects, as shown in Patient 1, it is generally better tolerated than conventional chemotherapy. This is crucial in a disease such as DT/AF, where curative options are scarce and the principal aim of the treatment is to improve quality of life. In addition, clinical benefit lasted for over one year in both cases and it is still ongoing. Fortunately, the relative lack of cumulative toxicity of pazopanib compared to standard chemotherapy allows an indeterminate duration of treatment.

In conclusion, pazopanib is a promising therapeutic option in DT/AF. However, owing to the retrospective nature of this report and the small number of patients this observation clearly needs to be confirmed in prospective studies. Thus, the French Sarcoma Group is currently conducting a phase II trial that assesses the efficacy and tolerance of pazopanib in DT/AF (ClinicalTrials.gov identifier NCT01876082). This encouraging novel strategy deserves further investigation.

## Consent

Written informed consent was obtained from the patients for publication of this Case Report and any accompanying images. Copies of the written consents are available for review by the Editor-in-Chief of this journal.

## Competing interests

The authors declare that they have no competing interests.

## Authors’ contributions

JML, CB, HM and IJ contributed to the conception, design and drafting of the manuscript. CM carried out the radiological evaluation. KT performed the histopathological analyses of the tumours. CB and IJ coordinated the manuscript drafting. All authors read and approved the final manuscript.

## References

[B1] Alman BA Pajerski ME Diaz-Cano S Corboy K Wolfe HJ Aggressive fibromatosis (desmoid tumor) is a monoclonal disorder Diagn Mol Pathol 1997 6 2 98 101 10.1097/00019606-199704000-00005 9098648

[B2] Biermann JS Desmoid tumors Curr Treat Options Oncol 2000 1 3 262 266 10.1007/s11864-000-0038-5 12057169

[B3] Bertagnolli MM Morgan JA Fletcher CD Raut CP Dileo P Gill RR Demetri GD George S Multimodality treatment of mesenteric desmoid tumours Eur J Cancer 2008 44 16 2404 2410 10.1016/j.ejca.2008.06.038 18706807

[B4] Bonvalot S Desai A Coppola S Le Péchoux C Terrier P Dômont J Le Cesne A The treatment of desmoid tumors: a stepwise clinical approach Ann Oncol 2012 23 Suppl 10 158 166 10.1093/annonc/mds298 22987953

[B5] Smith AJ Lewis JJ Merchant NB Leung DH Woodruff JM Brennan MF Surgical management of intra-abdominal desmoid tumours Br J Surg 2000 87 5 608 613 10.1046/j.1365-2168.2000.01400.x 10792318

[B6] Ballo MT Zagars GK Pollack A Pisters PW Pollack RA Desmoid tumor: prognostic factors and outcome after surgery, radiation therapy, or combined surgery and radiation therapy J Clin Oncol 1999 17 1 158 167 10458229 10.1200/JCO.1999.17.1.158

[B7] Hansmann A Adolph C Vogel T Unger A Moeslein G High-dose tamoxifen and sulindac as first-line treatment for desmoid tumors Cancer 2004 100 3 612 620 10.1002/cncr.11937 14745880

[B8] Deyrup AT Tretiakova M Montag AG Estrogen receptor-beta expression in extraabdominal fibromatoses: an analysis of 40 cases Cancer 2006 106 1 208 213 10.1002/cncr.21553 16333857

[B9] Constantinidou A Jones RL Scurr M Al-Muderis O Judson I Pegylated liposomal doxorubicin, an effective, well-tolerated treatment for refractory aggressive fibromatosis Eur J Cancer 2009 45 17 2930 2934 10.1016/j.ejca.2009.08.016 19767198

[B10] Heinrich MC McArthur GA Demetri GD Joensuu H Bono P Herrmann R Hirte H Cresta S Koslin DB Corless CL Dirnhofer S van Oosterom AT Nikolova Z Dimitrijevic S Fletcher JA Clinical and molecular studies of the effect of imatinib on advanced aggressive fibromatosis (desmoid tumor) J Clin Oncol 2006 24 7 1195 1203 10.1200/JCO.2005.04.0717 16505440

[B11] Penel N Le Cesne A Bui BN Perol D Brain EG Ray-Coquard I Guillemet C Chevreau C Cupissol D Chabaud S Jimenez M Duffaud F Piperno-Neumann S Mignot L Blay JY Imatinib for progressive and recurrent aggressive fibromatosis (desmoid tumors): an FNCLCC/French Sarcoma Group phase II trial with a long-term follow-up Ann Oncol 2011 22 2 452 457 10.1093/annonc/mdq341 20622000

[B12] Gounder MM Lefkowitz RA Keohan ML D'Adamo DR Hameed M Antonescu CR Singer S Stout K Ahn L Maki RG Activity of Sorafenib against desmoid tumor/deep fibromatosis Clin Cancer Res 2011 17 12 4082 4090 10.1158/1078-0432.CCR-10-3322 21447727 PMC3152981

[B13] Li M Cordon-Cardo C Gerald WL Rosai J Desmoid fibromatosis is a clonal process Hum Pathol 1996 27 9 939 943 10.1016/S0046-8177(96)90221-X 8816889

[B14] Martin-Liberal J Judson I Benson C Antiangiogenic approach in soft-tissue sarcomas Expert Rev Anticancer Ther 2013 13 8 975 982 10.1586/14737140.2013.820579 23944712

[B15] Potti A Ganti AK Tendulkar K Sholes K Chitajallu S Koch M Kargas S Determination of vascular endothelial growth factor (VEGF) overexpression in soft tissue sarcomas and the role of overexpression in leiomyosarcoma J Cancer Res Clin Oncol 2004 130 1 52 56 10.1007/s00432-003-0504-0 14600832 PMC12161778

[B16] Graeven U Andre N Achilles E Zornig C Schmiegel W Serum levels of vascular endothelial growth factor and basic fibroblast growth factor in patients with soft-tissue sarcoma J Cancer Res Clin Oncol 1999 125 10 577 581 10.1007/s004320050319 10473871 PMC12169192

[B17] Hayes AJ Mostyn-Jones A Koban MU A'Hern R Burton P Thomas JM Serum vascular endothelial growth factor as a tumour marker in soft tissue sarcoma Br J Surg 2004 91 2 242 247 10.1002/bjs.4398 14760675

[B18] Chao C Al-Saleem T Brooks JJ Rogatko A Kraybill WG Eisenberg B Vascular endothelial growth factor and soft tissue sarcomas: tumor expression correlates with grade Ann Surg Oncol 2001 8 3 260 267 10.1007/s10434-001-0260-9 11314944

[B19] Iyoda A Hiroshima K Baba M Fujisawa T Yusa T Ohwada H Expression of vascular endothelial growth factor in thoracic sarcomas Ann Thorac Surg 2001 71 5 1635 1639 10.1016/S0003-4975(01)02533-4 11383813

[B20] George D Platelet-derived growth factor receptors: a therapeutic target in solid tumors Semin Oncol 2001 28 5 Suppl 17 27 33 10.1053/sonc.2001.29185 11740804

[B21] Négrier S Raymond E Antiangiogenic treatments and mechanisms of action in renal cell carcinoma Invest New Drugs 2012 30 4 1791 1801 10.1007/s10637-011-9677-6 21573959

[B22] Savage DG Antman KH Imatinib mesylate–a new oral targeted therapy N Engl J Med 2002 346 9 683 693 10.1056/NEJMra013339 11870247

[B23] van der Graaf WT Blay JY Chawla SP Kim DW Bui-Nguyen B Casali PG Schöffski P Aglietta M Staddon AP Beppu Y Le Cesne A Gelderblom H Judson IR Araki N Ouali M Marreaud S Hodge R Dewji MR Coens C Demetri GD Fletcher CD Dei Tos AP Hohenberger P EORTC Soft Tissue and Bone Sarcoma Group Pazopanib for metastatic soft-tissue sarcoma (PALETTE): a randomised, double-blind, placebo-controlled phase 3 trial Lancet 2012 379 9829 1879 1886 10.1016/S0140-6736(12)60651-5 22595799

